# Supplementary material for: The Gut Bacteria Dysbiosis Contributes to Chronic Graft-Versus-Host Disease Associated With a Treg/Th1 Ratio Imbalance
Source: Front Microbiol. 2022 Sep 8;13:813576. doi: 10.3389/fmicb.2022.813576 (PMC9493085; doi:10.3389/fmicb.2022.813576)
Supplement: Supplementary file 1 [file Data_Sheet_1.zip › P101SC18090073-01-B1-3-4_result/04.BetaDiversity/PCoA/weighted_unifrac/PCoA12_with_cluster.pdf]

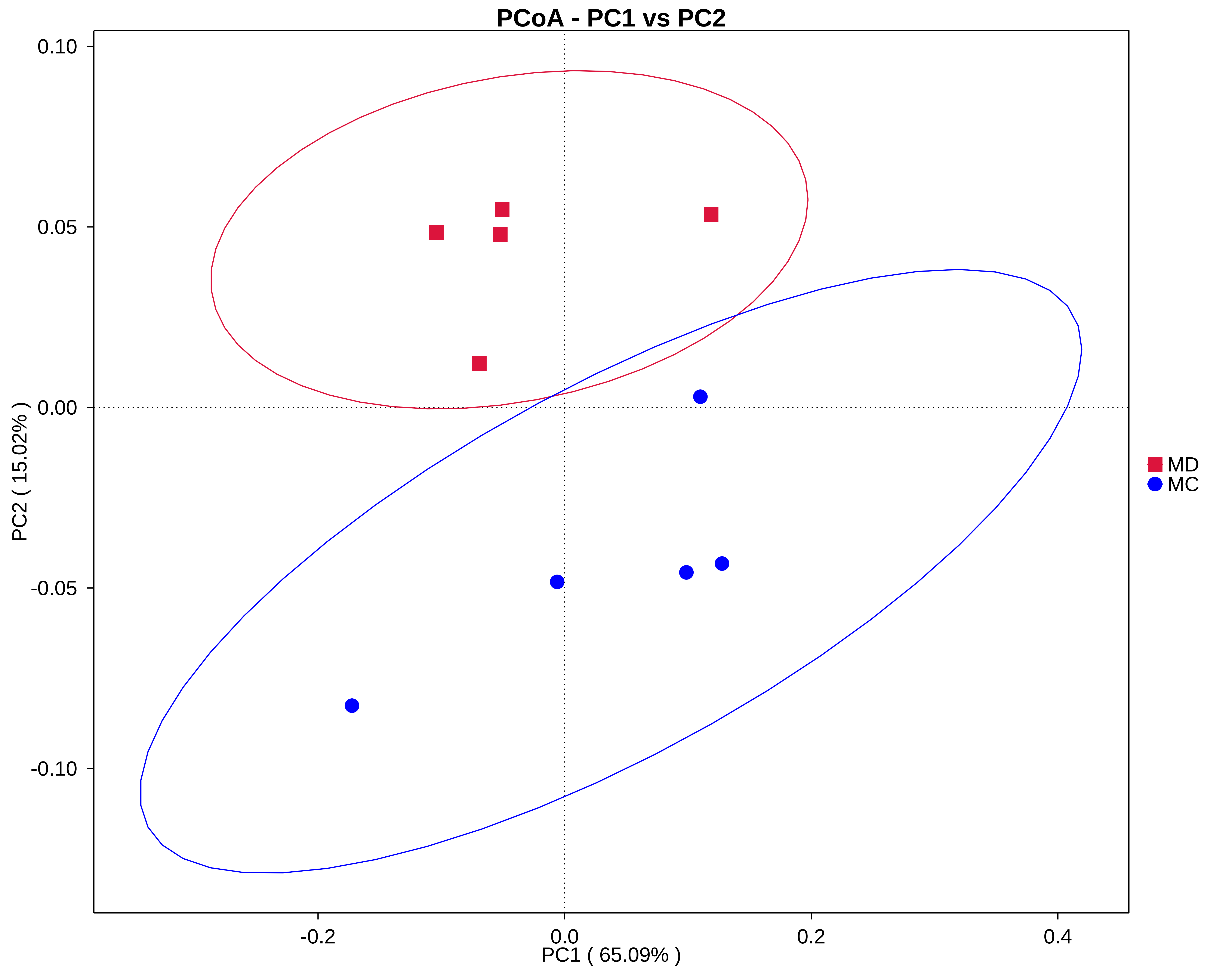
PCoA - PC1 vs PC2
0.10
0.05
0.00
-0.05
-0.10
PC2 (15.02%)
-0.2
0.0
0.2
0.4
PC1 (65.09%)
MD
MC
